# An Uncommon Location for a Urinary Tract Stone: Urethral Stone

**DOI:** 10.5334/jbsr.2922

**Published:** 2022-12-19

**Authors:** Anas Da’meh, Amin Da’meh

**Affiliations:** 1 Misitry of Health, JO; 2 UZ Brussel, BE

**Keywords:** Urolithiasis, flank pain, urethral stricture, hydronephrosis, ascending urethrogram, computed tomography

## Abstract

**Teaching point:** Attention to the urethra is essential when looking for urinary tract stones or etiology for hematuria.

## Case History

A 66-year-old male presented to the emergency department complaining of left flank pain increasing with time despite analgesics. The patient was treated for an obstructive lithiasis with a diameter of 12.5 mm at the level of left vesicoureteral junction.

ESWL was unsuccessful, and a ureterorenoscopy was performed. During this procedure, a urethral stricture was found, and an retrograde urethrogram showed a large urethral stone which was missed during the last radiological examinations (X-ray and CT).

**Figure 1:**
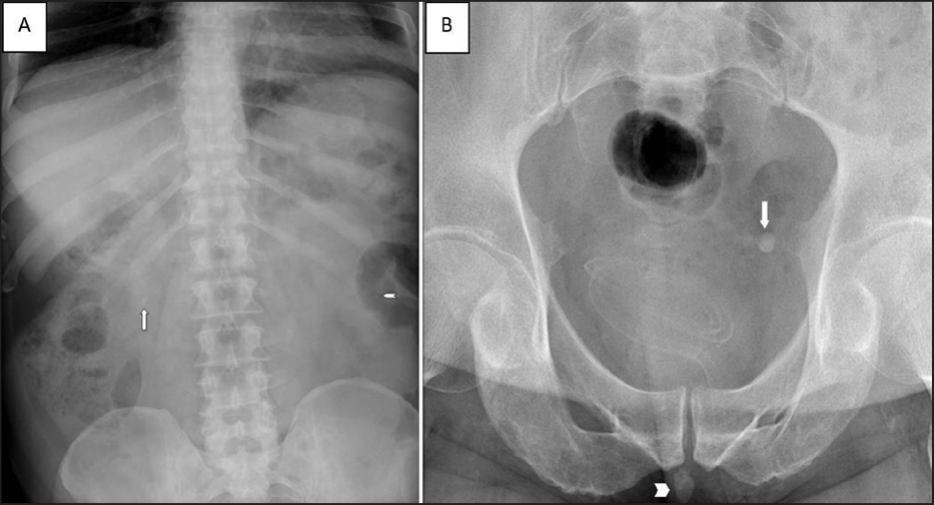


A previous abdominal X-ray showed multiple opacities in the area of the lower pole of the left kidney (Figure 1A, arrowhead), a single opaque stone in the area of the right kidney (arrow), and an opaque stone presumably in left vesicoureteral junction (Figure 1B, arrow). In addition, in retrospect a large opacity was seen projecting at the level of the posterior urethra (Figure 1B, arrowhead).

**Figure 2:**
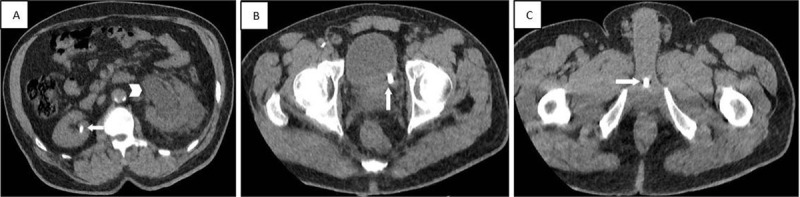


A previously performed CT confirmed the right renal stone (Figure 2A, arrow), a severe hydronephrosis (Figure 2A, arrowhead) of the left kidney due to distal obstructive ureteral stone (Figure 2B, arrow), and a stone at the level of the bulbous urethra (Figure 2C, arrow), which was initially overlooked.

**Figure 3:**
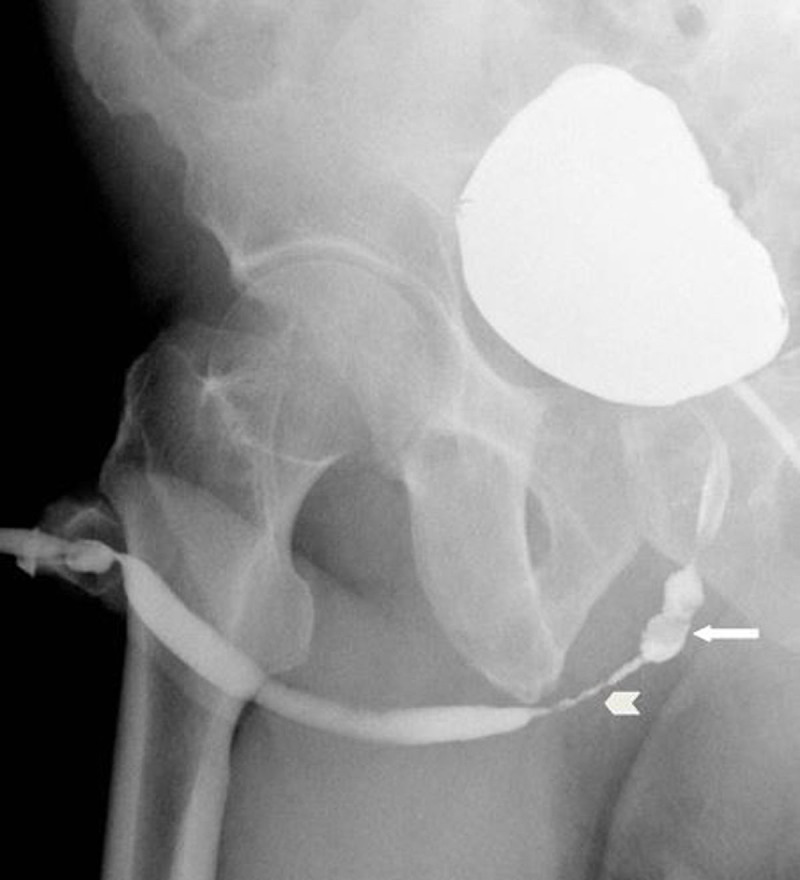


The retrograde urethrogram showed a filling defect in the bulbous urethra and (Figure 3, arrow) and a high grade stricture of approximately 3 cm length of the bulbous urethra (Figure 3, arrowhead).

## Comment

Urinary tract stones are most commonly located in the upper urinary tract. In rare cases (0.3–2%) they can be located in the urethra [1]. Urethral stones are more frequent in males because of specific anatomy and length.

Impaction of the stone usually occurs at the level of the prostatic urethra, although some authors reported higher occurrence in the anterior urethra or equal occurrence in the anterior and posterior urethra.

Urethral stones can be primary as they develop in or proximal to a urethral stricture, a diverticulum or a neurogenic bladder, or secondary, descending from the upper urinary system. Acute urinary retention is the usual clinical presentation of a urethral stone. However, urethral stones can cause pain and obstructive symptoms without urinary retention, depending on the size and location. Misdiagnosing urethral stones may lead to urethral damage, urethro-cutanous fistula, incontinence, hydronephrosis and obstructive kidney damage.

In conclusion, imaging has an important role in diagnosing a urethral stone, especially when patients present with non-specific complaints such as dysuria of various severity, hematuria or interruption of the urinary stream during micturition.
